# The Modulatory Effect of Ischemia and Reperfusion on Arginine Vasopressin-Induced Arterial Reactions

**DOI:** 10.1155/2016/3679048

**Published:** 2016-08-03

**Authors:** Katarzyna Szadujkis-Szadurska, Bartosz Malinowski, Małgorzata Piotrowska, Grzegorz Grześk, Michał Wiciński, Marta Gajdus

**Affiliations:** Department of Pharmacology and Therapeutics, Collegium Medicum Nicolaus Copernicus University, Sklodowskiej-Curie Street 9, 85-094 Bydgoszcz, Poland

## Abstract

*Aim of the Study.* The purpose of this study was to investigate the impact of ischemia and reperfusion on the resistance of arteries to AVP (arginine vasopressin), with a particular emphasis on the role of smooth muscle cells in the action of vasopressin receptors and the role of the cGMP-associated signalling pathway.* Materials and Methods.* Experiment was performed on the perfunded tail arteries from male Wistar rats. The constriction triggered by AVP after 30 minutes of ischemia and 30 and 90 minutes of reperfusion was analysed. Analogous experiments were also carried out in the presence of 8Br-cGMP.* Results.* Ischemia reduces and reperfusion increases in a time-dependent manner the arterial reaction to AVP. The presence of 8Br-cGMP causes a significant decrease of arterial reactivity under study conditions.* Conclusions.* Ischemia and reperfusion modulate arterial contraction triggered by AVP. The effect of 8Br-cGMP on reactions, induced by AVP after ischemia and reperfusion, indicates that signalling pathway associated with nitric oxide (NO) and cGMP regulates the tension of the vascular smooth muscle cells.

## 1. Introduction

Numerous mechanisms regulate arterial blood flow and tissue supply under various conditions. Metabolically active endothelial cells control blood pressure, tension and permeability of vessels, adhesion of inflammatory cells, and platelet aggregation. They are also considered initiators of reperfusion [1]. Diameter of arteries is changed by the endothelium; it results in the blood flow and perfusion changes in response to the vasoconstrictors (i.e., vasopressin) and vasodilators (i.e., nitric oxide) [2, 3]. Vascular smooth muscle cells are responsible for contractility, growth, and remodelling of blood vessels. Contraction of smooth muscle cells depends on the cytoplasmic calcium ions levels and the extracellular environment [3–6]. NO stimulates guanylate cyclase to produce cGMP, thus leading to relaxation of a vessel.

Blood vessel occlusion leads to ischemia. Cells have energy reserves and an ability to increase the amount of available oxygen, to survive a reduced blood supply. If ischemia lasts for longer period of time, changes in cellular structure occur with a possible necrosis [4, 7]. Studies indicated an inhibitory effect of ischemia on arterial contractility and shows clear association between NO synthesis and activation of cGMP [8, 9].

Hyperaemia is the first response to restored circulation along a given segment. During reperfusion, cellular defence mechanisms are overloaded, which may result in cellular damage [4]. That is why duration of ischemia is so important. The shorter it is, the less severe consequences for cells, tissues, and organs appear. Increased blood flow may cause physical and biochemical disorders in tissues, which may lead to reperfusion cascade, production of intracellular free radicals, release of cytokines, and inflammation development [10].

Therapy involving blockade of the initiators in the reperfusion cascade during the free radicals production is the most effective. That avoids both local and systemic disturbances. After that stage, a further therapy would be aimed at inhibition of neutrophils, granulocytes migration, and production of prostaglandins. However, if the inflammatory process and tissue damage are extensive enough to trigger the last part of the cascade, the therapy would be less effective [4, 11].

Studies on reperfusion in tail arteries of rats demonstrated increased contractile postreperfusion arterial reactions under an influence of an agonist. It was also mentioned that the reperfusion disturbed balance of the cellular antioxidative system. It was also suggested that the effect of cGMP is not limited to the relaxing action but also modulates reactions triggered by ROS [12].

VP is a nonapeptide of numerous functions. VP is synthesised by hypothalamus and accumulated in the posterior lobe of the pituitary gland. First studies in 1895 demonstrated the hormone's ability to constrict blood vessels, hence its name [13]. However, in 1956 it was demonstrated that VP is the same substance as the antidiuretic hormone (ADH), and both names are still in use as synonyms [14].

VP deficiencies occur in some shock states, but external administration of AVP may alleviate them. Studies have demonstrated that introduction of a low dose of VP in patients with dilated blood vessels reduced the required norepinephrine (noradrenaline) dose, making it applicable in case of septic shock. However, it was also paradoxically demonstrated that VP causes relaxation of some blood vessels which makes the hormone different from other vasoconstrictors [15].

Opinions concerning VP administration in cases of cardiac arrest are contradictory. On one hand, it was observed that VP injection during cardiopulmonary resuscitation directs blood from the skin, muscles, and intestines to the heart and brain, which has a favourable effect on function of those organs after restoration of circulation [16, 17]. However, the effect may be dangerous for the organism as a whole and hinder relaxation of the heart, whereas its contraction remains unchanged [18]. There were also studies indicating protective effect of VP on rat heart during the organ reperfusion after a long hypoxia [19].

There are also reports suggesting that VP may be secreted directly to the brain through an alternative route, acting as a neurotransmitter responsible for regulation of water-electrolyte homeostasis and production of cerebrospinal fluid [1, 20]. VP has also antipyretic properties. Its administration may even lead to hypothermia [21].

Metabotropic receptors V1a are present in large quantities in arterial smooth muscle cells and cause contraction of vessels. Those receptors are also abundant in myocytes, cerebral tissue, the superior cervical ganglion of the sympathetic trunk, the liver, blood cells, and the renal core. Precise role of VP in those tissues is subject to numerous studies all over the world [7, 20, 22, 23].

V1b occurs in the pituitary gland, cells of kidneys, brain, thymus, uterus, the myocardium, and lungs [24]. The V2 receptor is largely responsible for antidiuretic character of VP.

The purpose of this study was to investigate the effect of ischemia and reperfusion on reactions of resistance arteries on AVP, with a particular emphasis on the role of smooth muscle cells in the action of VP receptors, and to determine the role of the cGMP-associated signalling pathway in modulation of arterial reaction to AVP.

## 2. Materials and Methods

Our experiment was performed on isolated and perfused tail arteries collected from male Wistar rats under anaesthesia. Rats weighted 220–270 g.

After removal of surrounding tissues, the proximal section of the artery, approximately 3 cm long, was cannulated and, with the distal end attached to a 0.5 g weight, the artery was connected to the perfusion pressure recorder. The specimen was positioned vertically in a 20 mL container for isolated organs filled with aerated Krebs fluid, at 37°C and pH 7.4.

Ischemia was induced by clamping proximal fragment of the artery for 30 minutes. After that time, the artery was cut. In cases of reperfusion, the clamp was removed 30 minutes later, and the artery was cut free after another 30 or 90 minutes.

Krebs fluid, AVP (Sigma), and 8Br-cGMP (Behringer) were used in the study.

To determine the arterial reactivity to AVP, the study involved initial drawing of the control concentration-response curve (CRC), using van Rossum's method of accumulated concentrations [25] modified by Grześk and Szadujkis-Szadurski [26, 27]. Then, CRC in the presence of 8Br-cGMP was determined. Study agents were administered into the 20 mL container for isolated organs (extravascularly). Based on individual CRCs, the EC_50_ value (concentration triggering 50% of the maximum reaction) and the pEC_50_ value (−log⁡10 of EC_50_) were determined. In our experiments, contraction of the vessel was measured by increased pressure of perfusate in the experimental system, at the fixed flow of the fluid (approximately 1 mL/min).

The statistical analysis involved determination of mean values, standard deviations, and EC_50_ and pEC_50_ values. Statistical differences were calculated with the ANOVA test. Statistically significant values were interpreted as *p* < 0.05. Calculations were completed using the GraphPad Prism 5 software.

## 3. Results

**Figure 1:**
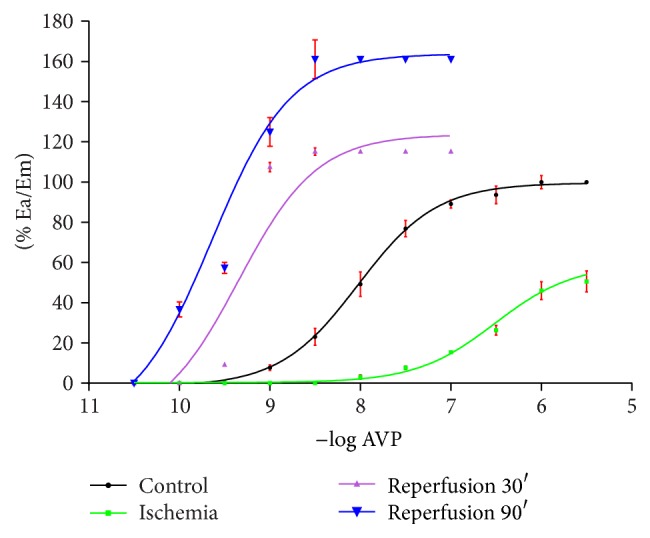
The effect (% Ea/Em) of ischemia 30′ and reperfusion 30′ and 90′ on CRCs for AVP.

AVP triggers the increase of perfusion pressure in a concentration-dependent manner. Influenced by 30-minute ischemia, the CRC is shifted to the right, with a simultaneous reduction of the maximum effect. After 30 and 90 minutes of reperfusion, CRCs are shifted to the left, with a simultaneous rise of the maximum effect, in the reperfusion time-dependent manner (Figure 1).

Noted changes of curve positions before and after reperfusion are statistically significant. The determined values % Ea/Em and EC_50_ for AVP in control conditions, and after ischemia and reperfusion, are presented in the Table 1.

**Figure 2:**
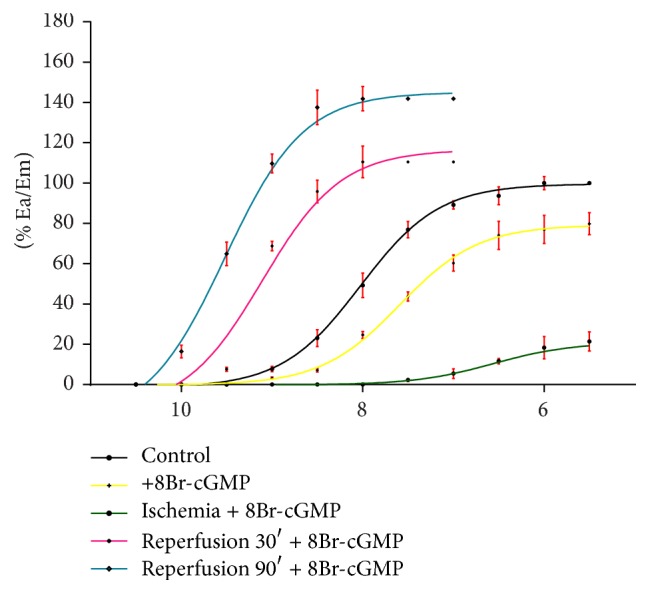
The effect (% Ea/Em) of 8Br-cGMP on CRCs for AVP in control, ischemia 30′, and reperfusion 30′ and 90′ conditions.

Another series of experiments was carried out in the presence of 0.01 mM/L of 8Br-cGMP, in order to check the role of the NO-regulated signalling pathway in AVP-triggered reactions of arteries. After addition of 8Br-cGMP, the CRC drawn for AVP was shifted to the right, with reduction of the maximum effect. After 30 minutes of ischemia, a further shift of the CRC to the right was observed, with reduction of Em. After 30 and 90 minutes of reperfusion with addition of 8Br-cGMP, statistically significant CRCs shift to the left was observed, in relation to the control (Figure 2, Table 2).

**Figure 3:**
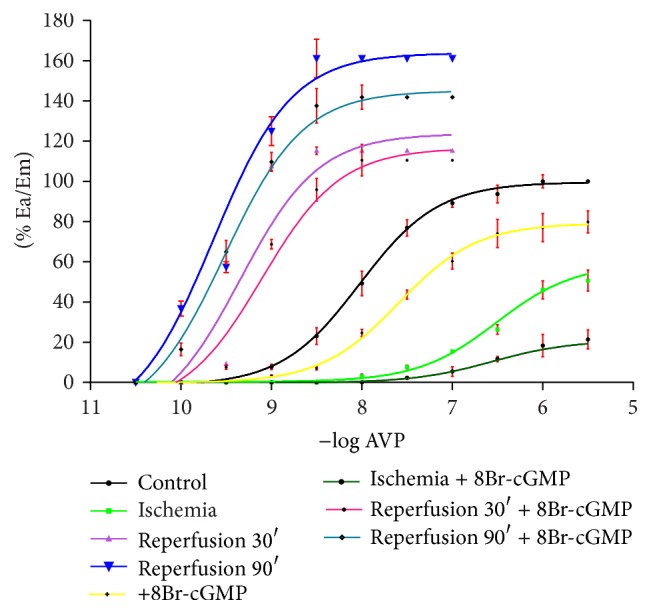
The effect (% Ea/Em) of ischemia 30′, reperfusion 30′ and 90′, and 8Br-cGMP added in those conditions on CRCs for AVP.

Comparison of CRCs drawn for AVP after I/R and in the presence of 8Br-cGMP demonstrates that the strongest arterial contractile reaction occurs after 90 minutes of reperfusion with no 8Br-cGMP. The lowest reaction is found for postischemia arteries with 8Br-cGMP (Figure 3).

Control arteries, after 90 minutes of reperfusion, after 30 minutes of reperfusion, and after ischemia with 8Br-cGMP, present their CRCs shifted to the right in relation to corresponding curves with no reagent added (Figure 3).

## 4. Discussion

Surgical procedures, including coronary artery bypass grafting or transplantations, are associated with temporary ischemia followed by reperfusion, which may lead to a local contraction of smooth muscles [28, 29]. Increased muscular contractility developing after I/R causes an extensive increase of calcium ion level and damage of endothelial cells and smooth muscles. That may disturb the equilibrium between factors causing constriction and relaxation of vessels and cause a complete elimination of blood flow [30, 31].

VP is one of the best known vasoconstrictors. VP is responsible for osmosis and cardiovascular homeostasis. In normal conditions, the level of VP ranges between 1 and 5 pg/mL. Numerous studies have been realised involving external administration of VP and leading to a significant rise of its concentration in the organism. Yang et al. [21] demonstrated, after their predecessors [32, 33], that endogenous VP causes a decrease of body temperature in rodents. Moreover, the receptor V1a, participating in that process [32], is the same receptor that is responsible for vasoconstrictive role of VP. It is interesting, as triggering an inflammatory reaction is one of the elements of the reperfusion cascade. Antipyretic properties of VP may influence the reaction of arteries after reperfusion, avoiding subsequent stages of the cascade. However, its excessively high level may be associated with a different effect, as intravenously and intracerebrally administered VP leads to hypothermia [21].

Studies on the role of AVP in brain injuries are also on their way. Experiments carried out by Szmydynger-Chodobska et al. demonstrated a pathological role of VP in brain injuries, including amplification of the posttraumatic production of proinflammatory mediators [23]. Manaenko et al. demonstrated that inhibition of the receptor V1a reduced brain injury and oedema [20].

Martikainen et al. [34] in their studies on swine model verified the role of VP after cerebral death with small intestine transplantation in patients with the short bowel syndrome. Cerebral death leads to increase of intracranial pressure, which gradually reduces blood flow to the brain. Cerebral ischemia causes activation of the sympathetic nervous system, which leads to narrowing of vessels and increased arterial blood pressure. However, in a short period of time the effect disappears, with resulting hypotension and hypoperfusion, which forces surgeons to apply vasoconstrictors [18, 34]. Despite the fact that vasopressin has reduced blood pressure, the systemic and enteral blood flow was endangered, and that circulation was of a particular interest considering the organ collected for transplantation. With VP, insufficient amounts of oxygen were supplied to tissues. That effect was not observed with other vasoconstrictors [34]. However, Rosendale et al. demonstrated a positive effect of pharmacological agents, including VP, on increased number of organs (heart) for transplantations [35].

In present experiments, AVP, a nonselective agonist of VP receptors type V1a, present in vascular smooth muscle cells, was used. It is a metabotropic receptor, activation of which leads to contraction of blood vessels as a consequence of inflow of calcium ions to the cytoplasm.

The pEC_50_ value of 7.987, determined for AVP in this study, is comparable to other studies in which arterial smooth muscle cells contraction was triggered with AVP (7.76 ± 0.14). The value is close to the one determined in studies on phenylephrine-triggered contraction of smooth muscle cells (pEC_50_ = 7.13 ± 0.06). Phenylephrine is a relatively selective agonist of the adrenergic receptor type *α*1. Similarly as in the case of AVP, contraction of the smooth muscle cells occurs with inflow of calcium ions to cytoplasm [36, 37]. Both receptors are activated in course of therapy of septic shock by administration of noradrenalin and dopamine (*α*-adrenergic receptor) followed by desmopressin (V1a) [36–38].

The effect of AVP on contraction of vascular smooth muscle cells was studied on arteries with preserved endothelium, being an integral part of the vascular wall, and regulating function of the vessel through release of various substances modulating tension of smooth muscle cells. If a vessel functions normally, endothelium reduces reactivity of the artery through release of prostanoids and NO. NO has a strong relaxing effect on the vessel. However, if endothelium is damaged, the vessel becomes more susceptible to stimulation with constrictors. A similar effect may be achieved by inhibition of NO synthase activity [39, 40].

8Br-cGMP is a cGMP analogue, that is hardly hydrolysed and may easily penetrate cells. Its action is analogous to the action of NO. Therefore, the mechanism of 8Br-cGMP corresponds to cGMP-dependent reactions occurring as a result of synthesis of NO. That leads to cellular hyporeactivity [9, 41]. Studies demonstrated a significant effect of 8Br-cGMP on arterial reactions to AVP. Values for the control after addition of the reagent (pEC_50_ = 7.587) are slightly higher than reported from another study for the same concentration (pEC_50_ = 6.94 ± 0.12) [12, 23], but both studies indicate the relaxing role of 8Br-cGMP in case of artery stimulation with AVP. Also the shape of CRCs compared to a common control is observed.

Similarly to other vasoconstrictors, ischemia caused a significant reduction of arterial reactions, demonstrating statistical significance at all levels of concentration for which the arterial reaction was observed. Addition of 8Br-cGMP reduced arterial contractility even more, exhibiting statistical significance for the last four concentrations of the administered AVP. NO is the best known substance acting through cGMP [42]. Reaction of cyclic nucleotides, including cAMP and cGMP, is converse to the amount of calcium ions in vascular smooth muscle cells. With decreased amount of [Ca^2+^] and with reduced smooth muscle cells susceptibility to those ions, nucleotide vasodilating action is increased [9].

A similar arterial reaction was demonstrated for other vasoconstrictors, including angiotensin II (ANG II) after 30 minutes of ischemia [39]. After that time, the EC_50_ value for arteries under the effect of AVP is 3.355 × 10^−6^ and for ANG II is 2.89 × 10^−7^. Although those values are not identical, the obtained concentration-result curves indicate similar action of both vasoconstrictors.

This study aimed at the effect of AVP on reaction of the smooth muscle cells under reperfusion conditions. Reperfusion lasting 90 minutes reached the highest ratio (% Ea/Em = 168.60). The EC_50_ value was 2.546 × 10^−9^. The result is comparable to those for ANG II (2.17 × 10^−9^). That indicates similarity of both vasoconstrictors under conditions of ischemia and reperfusion.

Similarly to the reperfusion lasting for 90 minutes, the 30-minute reperfusion triggered a more pronounced arterial reaction to AVP than the control. However, the reaction was less pronounced than the reaction of arteries after a 90-minute reperfusion. The shape of curves resembles curves drawn for ischemia and 30- and 90-minute reperfusion in relation to the control for ANG II in the analogous study.

The study demonstrated that CRCs for AVP in the presence of 8Br-cGMP are shifted to the right, with a simultaneous reduction of the maximum effect. Similarly to other studies, the presented results indicate unaltered effect of the compound on blood vessels. 8Br-cGMP (and consequently also NO) inhibits overreactivity of blood vessels to AVP triggered by reperfusion and reduced reaction of arteries for the control and ischemia [39]. Similarity of the reaction of the smooth muscle cells stimulated by AVP to the reaction of arteries under the influence of ANG II in a vessel with preserved endothelium allows presumption, that a similar effect would be observed for endothelium-depleted arteries. Contractive hyperreactivity after reperfusion achieved in the experiment, along with literature data, indicates that it is endothelium-independent. However, the relaxing effect of NO is observed [9, 31]. Excessive contraction of the artery leads to damage of its smooth muscle cells and endothelium, with longer reperfusion that leads to increased contraction.

## 5. Conclusions

Ischemia and reperfusion modulate arterial constriction triggered by AVP. The effect of 8Br-cGMP on reactions stimulated by AVP after ischemia and reperfusion indicates participation of the signalling pathway associated with NO and cGMP in regulation of the tension of the smooth muscle cells triggered by the vasoconstrictor.

## Figures and Tables

**Table 1:** % Ea/Em, EC_50_, and pEC_50_ values for AVP determined in control, after ischemia 30′, and after reperfusion 30′ and 90′ conditions.

	% Ea/Em	EC_50_	pEC_50_
Control	100,00	9,696 × 10^−7^	7,987
Ischemia	58,92	3,355 × 10^−6^^*∗*^	6,526^a^
Reperfusion 30′	124,50	1,535 × 10^−9^^*∗∗*^	9,186^b^
Reperfusion 90′	168,60	2,546 × 10^−9^^*∗∗∗*^	9,406^c^

EC_50_ control versus EC_50_^*∗*/*∗∗*/*∗∗∗*^, *p* < 0.05; pEC_50_ control versus pEC_50_^a/b/c^, *p* < 0.05; pEC_50_^b^ versus pEC_50_^c^, *p* = ns.

% Ea/Em: % of maximum reaction.

EC_50_: concentration triggering 50% of the maximum reaction.

pEC_50_: −log 10 of EC_50_.

**Table 2:** Values % Ea/Em, EC_50_, and pEC_50_ for AVP determined after ischemia and reperfusion in the presence of 8Br-cGMP.

	% Ea/Em	EC_50_	pEC_50_
Control	100,00	9,696 × 10^−7^	7,987
AVP + 8Br-cGMP	79,29	3,865 × 10^−7^^*∗*^	7,587^a^
Ischemia + 8Br-cGMP	21,03	3,492 × 10^−6^^*∗∗*^	6,543^b^
Reperfusion 30′ + 8Br-cGMP	117,70	7,583 × 10^−8^^†^	8,880^c^
Reperfusion 90′ + 8Br-cGMP	147,70	2,559 × 10^−9^^††^	9,408^d^

EC_50_ control versus EC_50_^*∗*/*∗∗*/†/††^, *p* < 0.05; pEC_50_ control versus pEC_50_^b/c/d^, *p* < 0.05; pEC_50_ control versus pEC_50_^a^, *p* = ns.

% Ea/Em: % of maximum reaction.

EC_50_: concentration triggering 50% of the maximum reaction.

pEC_50_: −log 10 of EC_50_.

## Abbreviations

ADH: Antidiuretic hormone
ANG II: Angiotensin II
AVP: Arginine vasopressin
CG: Guanylate cyclase
I/R: Ischemia/reperfusion
NO: Nitric oxide
ROS: Reactive oxygen species
VP: Vasopressin.
